# Body mass index trajectories in the first two years and subsequent childhood cardio-metabolic outcomes: a prospective multi-ethnic Asian cohort study

**DOI:** 10.1038/s41598-017-09046-y

**Published:** 2017-08-21

**Authors:** Izzuddin M. Aris, Ling-Wei Chen, Mya Thway Tint, Wei Wei Pang, Shu E. Soh, Seang-Mei Saw, Lynette Pei-Chi Shek, Kok-Hian Tan, Peter D. Gluckman, Yap-Seng Chong, Fabian Yap, Keith M. Godfrey, Michael S. Kramer, Yung Seng Lee

**Affiliations:** 1 Singapore Institute for Clinical Sciences, Agency for Science, Technology and Research, Singapore, Singapore; 2 Department of Paediatrics, Yong Loo Lin School of Medicine, National University of Singapore, Singapore, Singapore; 3 Department of Obstetrics and Gynaecology, Yong Loo Lin School of Medicine, National University of Singapore, Singapore, Singapore; 4 Saw Swee Hock School of Public Health, National University of Singapore, Singapore, Singapore; 5 Department of Obstetrics and Gynaecology, KK Women’s and Children’s Hospital, Singapore, Singapore; 6 Liggins Institute, University of Auckland, Auckland, New Zealand; 7 Department of Paediatrics, KK Women’s and Children’s Hospital, Singapore, Singapore; 8 Duke-NUS Medical School, Singapore, Singapore; 9 Lee Kong Chian School of Medicine, Nanyang Technological University, Singapore, Singapore; 10 MRC Lifecourse Epidemiology Unit and NIHR Southampton Biomedical Research Centre, University of Southampton and University Hospital Southampton NHS Foundation Trust, Singapore, Singapore; 11 Departments of Pediatrics and of Epidemiology, Biostatistics and Occupational Health, Faculty of Medicine, McGill University, Montreal, Canada; 12 Khoo Teck Puat-National University Children’s Medical Institute, National University Health System, Singapore, Singapore

## Abstract

We investigated body mass index (BMI) trajectories in the first 2 years of life in 1170 children from an Asian mother-offspring cohort in Singapore, and examined their predictors and associations with childhood cardio-metabolic risk measures at 5 years. Latent class growth mixture modelling analyses were performed to identify distinct BMI z-score (BMIz) trajectories. Four trajectories were identified: 73.2%(n = 857) of the children showed a normal BMIz trajectory, 13.2%(n = 155) a stable low-BMIz trajectory, 8.6%(n = 100) a stable high-BMIz trajectory and 5.0%(n = 58) a rapid BMIz gain after 3 months trajectory. Predictors of the stable high-BMIz and rapid BMIz gain trajectories were pre-pregnancy BMI, gestational weight gain, Malay and Indian ethnicity, while predictors of stable low-BMIz trajectory were preterm delivery and Indian ethnicity. At 5 years, children with stable high-BMIz or rapid BMIz gain trajectories had increased waist-to-height ratios [B(95%CI) 0.02(0.01,0.03) and 0.03(0.02,0.04)], sum of skinfolds [0.42(0.19,0.65) and 0.70(0.36,1.03)SD units], fat-mass index [0.97(0.32,1.63)SD units] and risk of obesity [relative risk 3.22(1.73,6.05) and 2.56 (1.19,5.53)], but not higher blood pressure. BMIz trajectories were more predictive of adiposity at 5 years than was BMIz at 2 years. Our findings on BMIz trajectories in the first 2 years suggest important ethnic-specific differences and impacts on later metabolic outcomes.

## Introduction

Childhood obesity is a major health concern worldwide^1^, because of its association with later cardio-metabolic outcomes such as coronary heart disease and Type 2 diabetes^2, 3^. These associations suggest that weight and body mass in early childhood may affect health risks in later life. Most epidemiological studies examining associations between childhood body mass index (BMI) and later cardio-metabolic outcomes have focused on BMI at only one time point^4, 5^. Childhood growth trajectory has recently been advocated as a predictor of future cardio-metabolic risk^6, 7^. Trajectory patterns account for dynamic changes in size that vary over time during the child’s development, providing an important dimension for consideration, in addition to just assessing size at one point in time.

Recent progress in statistical techniques makes it possible to study the potential heterogeneity of BMI changes in early childhood. Individual children may belong to distinct BMI trajectories^8, 9^ which may confer different risks towards the subsequent development of obesity or cardio-metabolic disease later in life. Techniques such as latent class growth mixture modelling (LCGMM) allow for estimation of such trajectories and their within-class variance, thereby allowing for greater heterogeneity in statistical model^10, 11^, unlike other trajectory models such as group-based trajectory modelling, which fix the within-class variation in each trajectory to zero^8^.

While many studies have prospectively explored BMI trajectories during childhood and adolescence^12–16^, few have examined BMI trajectories in the first 1000 days after conception (age 0 to 2 years)^17, 18^, which may be a sensitive window for the development (and hence potential prevention) of later obesity and cardio-metabolic disease^19, 20^. Even fewer studies have been conducted in Asian populations, whose susceptibility to metabolic disease often exceeds that in Western populations^21^. We are not aware of any accepted guidelines to identify clinically important weight gains^22^ or growth trajectories in children aged ≤2 years. Identifying groups of young children following trajectories associated with high risk of developing obesity or cardio-metabolic disease could potentially help in targeting early intervention. Using data from a prospective mother-offspring Asian cohort in Singapore, we aimed to identify distinct BMI trajectories in the first 2 years of life, and examine their predictors and their associations with cardio-metabolic risk measures at age 5 years. We also hypothesized that BMI trajectories in the first 2 years may be more predictive than static BMI measurement at 2 years.

## Methods

### Study population

The Growing Up in Singapore Towards healthy Outcomes (GUSTO) study has been previously described in detail^23^. Briefly, pregnant women were recruited in their first trimester at two major public hospitals in Singapore with obstetric services (KK Women’s and Children’s Hospital and the National University Hospital) between June 2009 and September 2010. Eligible women were Singapore citizens or permanent residents who were of Chinese, Malay, or Indian ethnicity with homogeneous parental ethnic backgrounds, and did not receive chemotherapy or psychotropic drugs and did not have diabetes mellitus. Of 3751 women approached, 2034 were eligible, 1247 were recruited and 1170 had singleton deliveries (Supplemental Fig. 1). The reasons of ineligibility have been previously described in detail^23^. Informed written consent was obtained from the women, and the study was approved by the National Healthcare Group Domain Specific Review Board and SingHealth Centralized Institutional Review Board. All methods were performed in accordance with the relevant guidelines and regulations, and ethical approval was granted by the National Healthcare Group Domain Specific Review Board and SingHealth Centralized Institutional Review Board.

### Maternal data

Socio-demographic data (age, self-reported ethnicity, educational attainment, income level and parity) were obtained at recruitment. Pregnant women underwent a 2-hour, 75-gram oral glucose tolerance test after an overnight fast at 26–28 weeks of gestation, as detailed previously^24^; those diagnosed with gestational diabetes based on World Health Organization’s (WHO) criteria [FPG ≥ 7.0 mmol/L or 2-hour glucose ≥7.8 mmol/L] were placed on a diet or treated with insulin. Gestational age (GA) was assessed by trained ultrasonographers at the first dating scan after recruitment and was reported in completed weeks.

Maternal pre-pregnancy weight was self-reported at study enrolment. Measurements of weight and height for mothers during pregnancy were obtained using SECA 803 Weighing Scale and SECA 213 Stadiometer (SECA Corp, Hamburg, Germany). These measurements were used to calculate body mass index (BMI) in kg/m^2^. Gestational weight gain (GWG) was calculated as the difference between last measured weight before delivery (between 35–37 weeks of gestation) and pre-pregnancy weight, and was corrected for gestational duration using maternal weight-gain-for-gestational age z-score charts by Hutcheon *et al*.^25^. Maternal blood pressure (BP) at 26–28 weeks of gestation was taken by trained research coordinators with an oscillometric device (MC3100, HealthSTATS International Pte Ltd, Singapore).

### Infant feeding

Mothers were asked about infant milk feeding using interviewer-administered questionnaires at home visits when the infants were 3, 6, 9, and 12 months of age. Feeding practices were classified into exclusive, predominant, and partial breastfeeding at each of those ages. Both direct breastfeeding and expressed breast milk intakes were classified as breastfeeding. Infants were defined as having low, intermediate or high breastfeeding, as detailed previously^26^.

### Child anthropometric measurements

Measurements of child weight and length/height were obtained at birth, 3, 6, 9, 12, 15 and 18 months and 2 years and 5 years of age, as detailed previously^27, 28^. At 5 years, we measured waist circumference, four skinfold thicknesses (triceps, biceps, subscapular and suprailiac), fat and lean mass [in a subset of children (n = 274) whose parents provided written consent] based on quantitative magnetic resonance imaging, as detailed previously^29^. These measurements were used to calculate BMI, sum of skinfolds (SSF), waist-to-height ratio (WHtR), fat mass index (FMI) and lean mass index (LMI) (calculated as fat or lean mass divided by square of height). Age- and sex-specific BMI z-scores (BMIz) were calculated using WHO refs 30, 31. Child obesity at 5 years was defined as age- and sex-specific BMIz two standard deviations higher than the median of the WHO ref. 31.

Based on standardized protocols^32^, child BP at 5 years was measured by trained research coordinators using a Dinamap CARESCAPE V100 (GE Healthcare, Milwaukee, WI), with the arm resting at the chest level. An average of two blood pressure readings were calculated if the difference between readings was <10 mmHg; otherwise, a third reading was taken and the average of the three readings used instead. Child prehypertension was defined as systolic (SBP) or diastolic (DBP) BP above the 90^th^ percentile for the child’s sex, age and height. As there is currently no reference for blood pressure percentiles in the Singapore population, we utilized reference values published by the American Academy of Pediatrics^33^.

### Statistical analysis

#### Stage 1: Modelling BMIz trajectories in the first 2 years using LCGMM

We analyzed child BMIz trajectories in the first 2 years of life using LCGMM^10, 11^. LCGMM is a longitudinal technique based on structural equation modelling that incorporates both continuous and categorical latent (unobserved) variables. The technique assumes that individuals in the sample need not come from a single underlying population, but rather from multiple, latent subgroups. Each identified subgroup has its own specific parameters (e.g., intercept, slope, quadratic), which are unobserved. Furthermore, LCGMM accounts for within-class variation in all growth parameters, implying within-class heterogeneity in addition to the between-class heterogeneity among the identified subgroups.

Quadratic-shaped trajectories were fitted, allowing for curved developmental patterns, with an increasing number of latent trajectories, assuming a constant variance–covariance structure (correlated random intercept, linear, quadratic function). The proportions of missing BMIz data at each time point and across all time points in the first 2 years are shown in Supplemental Table 1; 87.5% of children had at least 4 measurements of BMI in the first 2 years. We used the maximum likelihood robust estimator to account for missing data by full information maximum likelihood. This process approximates missing data by estimating a likelihood function for each individual based on variables that are present, such that all the available data points are used^34^. We identified the optimal number of latent trajectories based on two model-fit indices: the Bayesian Information Criterion (BIC) and the Bootstrap Likelihood Ratio Test (BLRT). A lower BIC value indicates a better model fit, while the BLRT provides a p-value indicating whether a model with one fewer trajectory groups (*k-1* model) should be rejected in favour of a model with *k* trajectories. Posterior probabilities of belonging to each trajectory were also examined, with subjects assigned to the trajectory for which they had the highest posterior probability. We required each trajectory to contain a minimum of 5% of subjects, so that it would be large enough to be clinically important. Distinct trajectories were coded as a categorical variable (with *k* number of categories) and were named based on their visual appearance. As the trajectories were similar in nature in both girls and boys (as found in other studies^13, 14^), all analyses were performed on the total sample. In addition, when corrected postnatal age for infants born preterm^35^ was used in deriving the trajectories, the patterns were exactly the same as those derived using uncorrected postnatal age. In light of this, all analyses were performed using uncorrected postnatal age. To illustrate the robustness of the extracted trajectories, we repeated the analyses restricted to children with no missing BMIz data in the first 2 years (n = 536). All LCGMM analyses were conducted using Mplus version 7.4 (Muthén and Muthén, Los Angeles, CA).

#### Stage 2: Predictors and cardio-metabolic consequences of BMIz trajectories

Associations between maternal (age, income level, pre-pregnancy BMI (ppBMI), height, GWG, GDM status, parity and GA at delivery) and infant (ethnicity and breastfeeding) factors and BMIz trajectories were first examined using ordinal logistic regression. However, the proportional odds assumption was violated (Brant test p < 0.05) for many of the predictors (ppBMI, height, GWG, income level, ethnicity and GA at delivery), rendering the model unsuitable for analysis and interpretation. We therefore used multinomial logistic regression, with the most commonly occurring trajectory chosen as the reference category. As self-reported pre-pregnancy weight may have limited validity, we also carried out sensitivity analyses by replacing ppBMI with maternal BMI at booking (mean 8.7 ± 2.8 weeks of gestation).

We studied the association between BMIz trajectories and cardio-metabolic measures at 5 years (i.e., WHtR, SSF, FMI, LMI, SBP and DBP) using multivariable linear regression. As the distributions of child WHtR, SSF, FMI and LMI were skewed, the data were log-transformed and standardized to z-scores with a mean 0 and SD of 1. The log-transformation reduced the skewness and the problem of non-normality. Poisson regression models with robust variance were used to calculate the relative risk of obesity or prehypertension at 5 years for each distinct BMIz trajectory.

For comparison, we also estimated the relative risk of obesity or prehypertension at 5 years for the (static) BMIz measurement at 2 years, categorized into four levels: <5^th^, 5^th^–<85^th^, 85^th^–<95^th^ or ≥95^th^ percentiles and compared those adjusted relative risk estimates to those associated with BMIz trajectories. To assess the variance of continuous cardio-metabolic outcomes explained by trajectory vs static BMIz groupings beyond baseline covariates, a basic model was first fitted by including predictors of cardio-metabolic outcomes (maternal income level, ppBMI, height, GWG, parity, GA at delivery, breastfeeding, child ethnicity, and sex) as independent variables in a linear regression analysis. Subsequently, the trajectory or static BMIz groupings were added separately to those baseline models; the increment in variance explained beyond that of baseline covariates was assessed using the adjusted R^2^ values. The area under the receiver operating characteristics (ROC) curve was also used to compare the predictive value of trajectory vs static BMIz groupings for obesity and prehypertension at 5 years.

All models were adjusted for maternal income level, ppBMI, GWG, parity, GA at delivery, breastfeeding, child ethnicity, and sex to reduce confounding, and exact age at measurement to improve precision. Recent studies have found a relationship between maternal height and offspring adiposity in childhood^36^; therefore, we also considered maternal height as a potential confounding variable in the analysis. Multiple imputation was used to account for missing covariates (maternal income level, n = 77; ppBMI, n = 105; height, n = 27; GWG, n = 33; breastfeeding, n = 142) with 20 imputations based on the Markov-chain Monte Carlo technique, using MI IMPUTE to impute the missing values and MI ESTIMATE to analyze the imputed datasets. These analyses were performed using Stata 13 software (StataCorp LP, TX).

### Data availability

Data are available from the National University of Singapore LORIS Database for researchers who meet the criteria for access to confidential data.

## Results

### BMIz trajectories in the first 2 years

**Figure 1:**
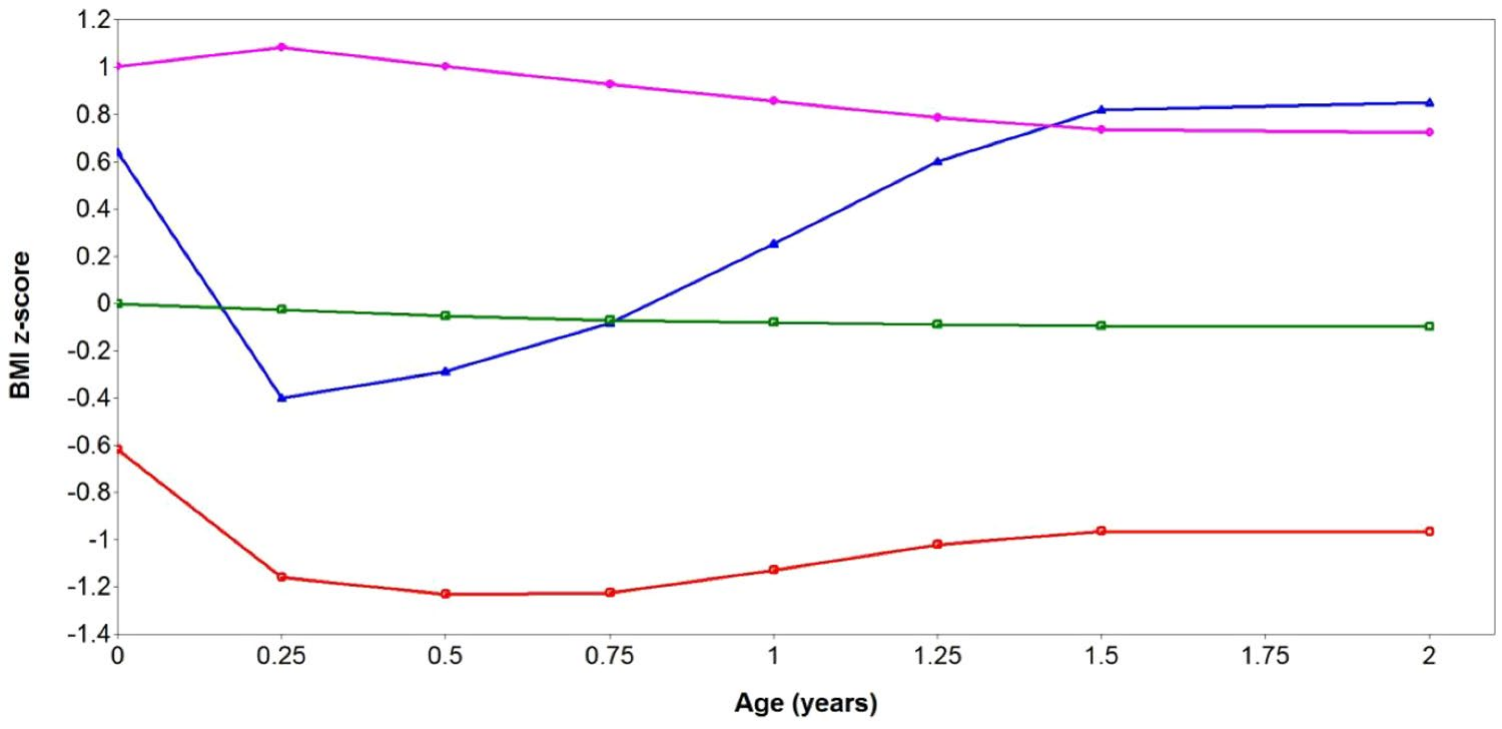
BMIz trajectories in the first 2 years of life in the GUSTO cohort. Red line = stable low BMIz trajectory (n = 155); Green line = Normal BMIz trajectory (n = 857); Purple line = stable high BMIz trajectory (n = 100); Blue line = Rapid BMIz gain after 3 months trajectory (n = 58). Values indicate mean BMIz at each time point.

Based on the BIC, BLRT and posterior probabilities, the “best-fitting model” (lowest BIC, significant BLRT p-value and posterior probability ≥0.70 for each subgroup) was the four-class model; the fit information indices are presented in Supplemental Table 2. Table 1 describes the demographic and clinical characteristics separately by BMIz trajectories. A large majority of the children (73.2%, n = 857) exhibited a normal BMIz trajectory, centred on BMIz = 0. The other three trajectories had distinct shapes: 13.2% (n = 155) had a stable low BMIz trajectory (average BMIz = −1 SD), 8.6% (n = 100) exhibited a stable high BMIz trajectory (average BMIz =  + 1SD) and 5.0% (n = 58) showed a rapid BMIz gain after 3 months trajectory in the first 2 years of life (Fig. 1). Sensitivity analyses using subjects with no missing BMIz data in the first 2 years (n = 536) showed the same trajectory patterns, further illustrating the robustness of the extracted BMIz trajectories (Supplemental Figure 2). Amongst children with no missing BMIz data, cross-tabulation analyses showed similar group assignments as in the full dataset (Supplemental Table 3). Supplemental Table 4 shows the corresponding percentiles at 2 years for each BMIz trajectory group; these percentiles closely approximate the thresholds based on standard categories (i.e., 5th, 50th, 85th and 95th percentiles).

**Table 1:** Demographic and clinical characteristics according to BMI z-score trajectories.

	All trajectories n = 1170	Stable low BMIz n = 155	Normal BMIz n = 857	Stable high BMIz n = 100	Rapid BMIz gain after 3 months n = 58	p value^c^
***Maternal characteristics***						
**Age (years)**	30.7 ± 5.1^a^	30.7 ± 5.0	30.6 ± 5.1	30.9 ± 5.3	31.1 ± 5.4	0.92
**Educational attainment**						0.58
<12 years	467 (40.5)^b^	65 (13.9)^c^	333 (71.3)^c^	41 (8.8)^c^	28 (6.0)^c^	
≥12 years	668 (50.5)	88 (12.8)	511 (74.3)	59 (8.6)	30 (4.3)	
**Income level per month**						0.03
<SGD $2000	165 (15.2)	24 (14.6)	121 (73.3)	8 (4.8)	12 (7.3)	
SGD $2000–5999	608 (55.6)	89 (14.6)	429 (70.6)	54 (8.9)	36 (5.9)	
≥SGD $6000	320 (29.2)	34 (10.6)	249 (77.8)	29 (9.1)	8 (2.5)	
**Parity**						0.08
Primiparous	534 (45.6)	61 (11.4)	409 (76.6)	37 (6.9)	27 (5.1)	
Multiparous	636 (54.4)	94 (14.8)	448 (70.4)	63 (9.9)	31 (4.9)	
**Pre-pregnancy BMI** ^**d**^ **(kg/m** ^**2**^ **)**	22.7 ± 4.4	22.2 ± 4.2	22.6 ± 4.3	24.1 ± 4.4	25.1 ± 5.6	<0.001
**Height (cm)**	158.3 ± 5.6	157.0 ± 5.3	158.4 ± 5.7	159.1 ± 5.4	157.7 ± 5.6	0.01
**GWG** ^**d**^ **z-score**	−1.01 ± 1.08	−1.29 ± 1.23	−1.00 ± 1.06	−0.73 ± 0.98	−0.95 ± 0.99	<0.001
**Gestational diabetes**						0.22
No	877 (81.1)	110 (12.5)	654 (74.6)	74 (8.4)	39 (4.4)	
Yes	204 (18.9)	34 (16.7)	138 (67.7)	18 (8.8)	14 (6.9)	
**Gestational age at delivery**						0.008
Term	1081 (92.3)	133 (12.3)	803 (74.3)	94 (8.7)	51 (4.7)	
Pre-term	89 (7.7)	22 (24.7)	54 (60.7)	6 (6.7)	7 (7.8)	
***Child characteristics***						<0.001
**Sex**						
Male	619 (52.9)	72 (13.1)	431 (78.2)	74 (11.9)	36 (5.8)	
Female	551 (47.1)	83 (13.4)	426 (68.8)	26 (4.7)	22 (4.0)	
**Ethnicity**						<0.001
Chinese	660 (56.4)	78 (11.8)	514 (77.9)	57 (8.6)	11 (1.7)	
Malay	298 (25.5)	27 (9.1)	213 (71.5)	35 (11.7)	23 (7.7)	
Indian	212 (18.1)	50 (23.6)	130 (61.3)	8 (3.8)	24 (11.3)	
**Breastfeeding type**						0.03
Low	461 (44.8)	71 (15.4)	321 (69.6)	34 (7.4)	35 (7.6)	
Intermediate	446 (43.4)	56 (12.6)	329 (73.8)	49 (10.9)	12 (2.7)	
High	122 (11.8)	15 (12.3)	90 (73.8)	10 (8.2)	7 (5.7)	

^a^Mean ±SD. ^b^n (%); indicates column percentages. ^c^n (%); indicates row percentages. ^c^Based on one-way ANOVA (for continuous variables) or chi-square test (for categorical variables). ^d^Abbreviations: BMI = body mass index; GWG = gestational weight gain.

### Predictors of BMIz trajectories in the first 2 years

The likelihood ratio test statistic of the multinomial logistic regression model yielded a p value of <0.01, indicating a significant association of the combined predictors with BMIz trajectory outcomes. Children born preterm [odds ratio (95% CI): 2.23 (1.28–3.36)] and of Indian ethnicity [2.36 (1.54–3.63) vs Chinese ethnicity] were more likely to be in the stable low BMIz trajectory. Children of Malay [3.49 (1.48–8.25)] and Indian ethnicity [6.30 (2.66–14.73)] were more likely to be in the rapid BMIz gain trajectory than those of Chinese ethnicity, while those born to multiparous mothers were more likely to be in the stable high BMIz trajectory [1.67 (1.07–2.61)] vs primiparous mothers. A 1-SD increase in maternal ppBMI or GWG was associated with a higher likelihood of belonging to the rapid BMIz gain and stable high BMIz trajectories, and a lower likelihood of belonging to the stable low BMIz trajectory (Table 2). Similarly, a 1-SD increase in maternal height was associated with a lower likelihood [0.79 (0.66–0.94)] of her child’s being in the stable low BMIz trajectory. Sensitivity analyses showed booking BMI, in place of ppBMI, was also a significant predictor of BMIz trajectories in the first 2 years (Supplemental Table 5).

**Table 2:** Predictors of BMI z-score trajectory groups in the first 2 years of life.

	Stable low BMIz			Stable high BMIz			Rapid BMIz gain after 3 months		
	Odds ratio^a^	95% CI		Odds ratio^a^	95% CI		Odds ratio^a^	95% CI	
		Low	High		Low	High		Low	High
**Income level (per month)** ^**b**^									
<SGD $2000	0.87	0.52	0.69	0.64	0.31	1.34	0.96	0.43	2.14
SGD $2000–5999	1.00	—	—	1.00	—	—	1.00	—	—
≥SGD $6000	0.66	0.42	1.03	1.17	0.69	1.99	0.41	0.15	1.12
**Parity** ^**b**^									
Primiparous	1.00	—	—	1.00	—	—	1.00	—	—
Multiparous	1.22	0.84	1.75	1.67	1.07	2.61	0.89	0.47	1.65
**Ethnicity** ^**b**^									
Chinese	1.00	—	—	1.00	—	—	1.00	—	—
Malay	0.71	0.44	1.17	1.51	0.90	2.53	3.49	1.48	8.25
Indian	2.36	1.54	3.63	0.57	0.27	1.21	6.30	2.66	14.73
**Gestational age at delivery** ^**b**^									
Term	1.00	—	—	1.00	—	—	1.00	—	—
Pre-term	2.23	1.28	3.86	1.46	0.66	3.22	1.51	0.51	4.53
**Pre-pregnancy BMI** ^**b**^									
**(per 1**SD **increase)**	0.73	0.59	0.91	1.49	1.19	1.88	1.43	1.06	1.95
**Height** ^**b**^									
**(per 1**SD **increase)**	0.79	0.66	0.94	1.08	0.87	1.34	0.94	0.68	1.28
**GWG z-score** ^**b**^									
**(per 1**SD **increase)**	0.83	0.69	0.99	1.51	1.19	1.90	1.39	1.02	1.90
**Breastfeeding type** ^**b**^									
Low	1.31	0.86	1.99	0.68	0.41	1.13	1.95	0.93	4.10
Intermediate	1.00	—	—	1.00	—	—	1.00	—	—
High	1.03	0.54	1.97	0.91	0.45	1.84	1.22	0.32	4.62

^a^Odds ratio estimates are referenced to the normal BMI z-score trajectory. ^b^All predictor variables are simultaneously included in the regression model.

#### Associations of early BMIz trajectories with later (5 years) cardio-metabolic measures

After adjusting for potential confounders, the stable low BMIz trajectory was significantly associated with lower WHtR [β (95% CI): −0.02SD units (−0.03, −0.01)], SSF [−0.43SD units (−0.62, −0.24)], FMI [−0.55SD units (−0.94, −0.15)], LMI [−0.68SD units (−1.07, −0.29)], SBP [−2.86 mmHg (−4.92, −0.80)] and DBP [−1.61 mmHg (−3.01, −0.20)], compared to the normal BMIz trajectory. Both the stable high BMIz and rapid BMIz gain trajectories were associated with higher WHtR and SSF, but not with SBP and DBP, compared to the normal BMIz trajectory (Table 3). Only the rapid BMIz gain trajectory was associated with higher FMI [0.97SD units (0.32, 1.63)], and only the stable high BMIz trajectory was associated with higher LMI [0.45SD units (0.05, 0.86)]. In addition, children in the rapid BMIz gain trajectory had significantly higher adiposity (WHtR, SSF, FMI) at 5 years compared to children in the stable high BMIz trajectory (Supplemental Table 6). Compared to the normal BMIz trajectory, both stable high BMIz [relative risk (95% CI): 3.22 (1.73, 6.05)] and rapid BMIz gain [2.56 (1.19–5.53)] trajectories were associated with an increased risk of obesity, while the stable low BMIz trajectory was associated with a decreased risk of obesity [0.12 (0.02, 0.85)] at 5 years. No significant associations were observed between early BMIz trajectories and later prehypertension (Table 4).

**Table 3:** Associations between BMI z-score trajectory subgroups with cardio-metabolic outcomes at 5-years.

BMI z-score trajectory	Stable low BMIz		Stable high BMIz		Rapid BMIz gain after 3 months	
Cardio-metabolic outcomes	β coefficient^a^	95% CI	β coefficient^a^	95% CI	β coefficient^a^	95% CI
Waist-to-Height Ratio^b^ (n = 864)	−0.02	−0.03, −0.01	0.02	0.01, 0.03	0.03	0.02, 0.04
Sum of skinfolds^b^ (n = 820)	−0.43	−0.62, −0.24	0.42	0.19, 0.65	0.70	0.36, 1.03
Fat-mass index^b^ (n = 247)	−0.55	−0.94, −0.15	0.35	−0.06, 0.75	0.97	0.32, 1.63
Lean-mass index^b^ (n = 247)	−0.68	−1.07, −0.29	0.45	0.05, 0.86	0.62	−0.02, 1.27
Systolic blood pressure^c^ (n = 757)	−2.86	−4.92, −0.80	0.83	−2.75, 4.42	1.49	−0.99, 3.96
Diastolic blood pressure^c^ (n = 757)	−1.61	−3.01, −0.20	−0.91	−3.35, 1.53	0.41	−1.27, 2.10

^a^β-coefficient estimates are referenced to the normal BMI z-score trajectory. ^b^Adjusted for maternal income level, ppBMI, height, GWG, parity, GA at delivery, breastfeeding, child ethnicity, sex and exact age at measurement. ^c^Adjusted for maternal income level, ppBMI, height, GWG, parity, GA at delivery, breastfeeding, blood pressure at 26–28 weeks of gestation, child ethnicity, sex and exact age at measurement.

**Table 4:** Relative risk and 95% CI of child obesity and prehypertension at 5-years according to BMIz trajectories and static BMIz at 2 years.

	Obesity at 5-years^a^ n = 65/870			Prehypertension at 5-years^b^ n = 92/757		
	Relative risk	95% CI		Relative risk	95% CI	
		Low	High		Low	High
**BMIz trajectories**						
Stable low BMIz (n = 155)	0.12	0.02	0.85	0.84	0.41	1.70
Normal BMIz trajectory (n = 857)	1.00	—	—	1.00	—	—
Stable high BMIz (n = 100)	3.22	1.73	6.05	1.23	0.60	2.53
Rapid BMIz gain (n = 58)	2.56	1.19	5.53	1.48	0.57	3.86
**Static BMIz at 2 years**						
< 5th percentile (n = 54)	0.68	0.09	4.90	0.90	0.41	1.94
5th–<85th percentile (n = 936)	1.00	—	—	1.00	—	—
85th–<95th percentile (n = 121)	1.79	0.82	3.90	1.03	0.51	3.19
≥95th percentile (n = 60)	6.96	3.67	13.20	1.24	0.48	3.19

^a^Adjusted for maternal income level, ppBMI, height, GWG, parity, GA at delivery, breastfeeding and child ethnicity. ^b^Adjusted for maternal income level, ppBMI, height, GWG, parity, GA at delivery, breastfeeding, blood pressure at 26–28 weeks of gestation and child ethnicity.

### Comparing early BMIz trajectories and static BMIz in predicting cardio-metabolic measures at 5 years

Early BMIz trajectory was more predictive of body composition measures (FMI and LMI) and obesity at 5 years than was (static) BMIz at 2 years. For FMI and LMI, the adjusted R^2^ values were 26.5% and 17.6%, respectively, in fully-adjusted models with BMIz trajectories; these R^2^ values were higher than those for static BMIz at 2 years (16.9% and 14.6%, respectively) (Supplemental Table 7). Furthermore, all BMIz trajectories in the first 2 years (compared to the normal BMIz trajectory) were significantly associated with obesity at 5 years (a negative association for the stable low BMIz trajectory and positive associations for the stable high BMIz and rapid BMIz gain trajectories), while BMIz ≥95^th^ percentile at 2 years (compared to BMIz 5^th^ – 85^th^ percentile) was the only static measure found to be significantly associated with obesity at 5 years [relative risk (95% CI): 6.96 (3.67–13.20)] **(**Table 4). The area under the ROC curve was also higher in fully-adjusted models for BMIz trajectories, compared to those for static BMIz at 2 years (Supplemental Table 7).

## Discussion

We have identified four distinct BMIz trajectories in the first 2 years of life in a multi-ethnic cohort of Asian children. We observed ethnic-specific differences and several factors predictive of the BMIz trajectories. Our findings suggest that different BMIz trajectories in the first 2 years reflect differential changes in body composition (fat vs lean mass) and are more predictive of body composition and obesity in later childhood than a single time point assessment of BMIz at 2 years.

The trajectory patterns observed in our study are consistent with those reported in previous studies by Magee *et al*.^13^ and Ventura *et al*.^15^, which also identified four BMI trajectories in childhood using LCGMM. The combined findings thus suggest that these four distinct BMI trajectories are real, rather than an artefact of the data-driven nature of the LCGMM technique. Other studies by Kwon *et al*.^18^ and Giles *et al*.^17^ also identified four BMI trajectories with similar patterns; those studies utilized a different technique known as group-based trajectory modelling, however, which assumes no within-class variation (i.e., variance in each subgroup is fixed to zero)^8^. LCGMM allows for greater heterogeneity because of its estimation of within-class variance for each trajectory^10, 11^.

The comparative strength and limitations of the LCGMM method over other alternative approaches (e.g., tracing growth on a growth chart) are the subject of some debate^37^. Clinicians often follow a “rule-of-thumb” method, in which excessive growth is indicated by crossing major percentile lines on a standard growth chart^38^. That method is simple and straightforward to implement, while LCGMM is relatively computer-intensive. The choice of the correct model and number of classes in LCGMM is also not always straightforward. The “rule-of-thumb” method however, assumes growth to be a linear function of size at different ages, which could subject the observed associations to statistical artefacts. LCGMM is capable of modelling non-linear growth curves, estimating individual trajectories and identifying distinctive subgroups in the population^37^. We believe these advantages outweigh its relative complexity.

We found that maternal adiposity-related factors (ppBMI, GWG) were associated with BMIz trajectories reflecting larger child size (stable high-weight and rapid weight gain). The positive relation between maternal adiposity-related factors and child size has been reported in other populations^39, 40^, as well as in our own cohort^27, 41^. Our findings provide further evidence that over-nutrition *in utero* may lead to high-risk BMI trajectories during early childhood. Although the mechanism is still not well-understood, it is believed that this may occur through increased transfer of maternal energy substrates, such as glucose, lipids and amino acids to the fetus^28, 42^, with the combined increase in all fuels likely contributing to the development of high-risk trajectories during early childhood^28^. We did not find an association between breastfeeding and the BMIz trajectories, consistent with earlier studies by Pryor *et al*.^14^, Giles *et al*.^17^ and Garden *et al*.^43^. While meta-analyses have suggested that breastfeeding is associated with a reduced risk of childhood overweight and obesity, this evidence is largely based on observational studies; reverse causality and residual confounding by behavioral or socio-economic attributes may explain the results of those studies.

We also observed ethnic differences in BMIz trajectories, with Malay children more likely than Chinese children to have rapid BMIz gain trajectories and Indian children more likely to have stable low BMIz or rapid BMIz gain trajectories. We have previously described that Indian children had smaller birth sizes and lower BMI in infancy compared to Chinese children^28^; this may potentially explain the relationship between Indian ethnicity and a low BMIz trajectory, which is the result of BMI tracking. Similar ethnic differences in rapid BMI gain trajectories have also been reported between African-American and white children in the U.S^12^. We have no clear biological explanation for the observed ethnic differences in rapid BMIz gain trajectories; an interplay between genetic and epigenetic factors, as well as a shared obesogenic environment, may be operating.

Maintenance of a stable high BMIz trajectory or accelerated BMIz gain was strongly associated with higher adiposity and increased obesity risk, while maintenance of a stable low BMIz trajectory protected against increased adiposity and obesity risk at 5 years, in line with earlier findings^17, 18^. This relationship may potentially be explained by BMI tracking, which has been demonstrated from infancy to middle childhood^44^. In addition, all BMIz trajectories in the first 2 years were significantly associated with obesity at 5 years (a negative association for stable low BMIz trajectory and positive associations for stable high BMIz and rapid BMIz gain trajectories), while BMIz ≥95^th^ percentile at 2 years was the only static measure with a significant association. A drawback of previous studies is the utilization of BMI at a single time-point as a predictor of subsequent obesity and cardiovascular risk^4, 5^. Such static assessments of BMI provide a snapshot of the “intensity” of adiposity, while ignoring the dynamics of BMI over time, such as duration, age of onset, and rate of rise of adiposity, which may have profound effects. Our findings provide evidence that both duration (reflected by stable high BMIz and stable low BMIz trajectories) and age of onset (reflected by rapid BMIz gain trajectory) of excess adiposity may be important factors in predicting subsequent cardio-metabolic risk. Our findings suggest that identification of developmental BMIz trajectories in the first 2 years may be helpful in identifying high-risk groups early in life, and thus in tailoring preventive interventions.

Strengths of our study include its prospective design, which is crucial for assessing the predictors of BMIz trajectories and their associations with cardio-metabolic measures later in childhood. Given the paucity of data in Asian populations on growth trajectories and their relation to later health outcomes, our findings fill an important gap and provide new insights into the role of early BMI development and its potential contribution to future metabolic disease risk in Asian populations. Our study also included several measures of BMI in the first 2 years, providing greater granularity in estimating BMIz trajectories, as well as several measures of adiposity at age 5 years, including WHtR, skinfolds and fat mass.

Limitations include the fact that maternal pre-pregnancy weight was self-reported at study enrolment, which may be affected by errors in recall. However, our data showed a strong correlation between self-reported pre-pregnancy weight and measured booking weight (ρ = 0.96), and sensitivity analyses using booking BMI as a predictor showed similar observations. Maternal smoking during pregnancy was not included as a predictor, owing to low prevalence in our study sample (2%), but was unrelated to child adiposity in previous analyses^45^. We were also unable to account for childhood dietary patterns or physical activity at 5 years, which may reflect exposure to an obesogenic environment. Half of the women approached were not eligible for inclusion in the cohort, and we had no wish to generalize our findings to ineligible women and children. We have previously shown that the ethnic background of those recruited and not recruited differed between these two groups, reflecting our *a priori* plans to recruit disproportionately from the minority ethnic groups^23^. Moreover, the strong associations of the stable high BMIz and rapid BMIz gain trajectories with increased adiposity and obesity risk observed in our study were in line with recent findings^17, 18^, also suggesting the robustness of our study findings. Finally, our study did not measure other cardio-metabolic biomarkers such as blood glucose, insulin, lipids, triglycerides and C-peptide, and thus lacking outcomes such as dysglycemia or dyslipidemia. In future studies, data on these outcomes would help clarify the health implications of our findings.

In conclusion, BMIz trajectories in the first 2 years were associated with cardio-metabolic measures later in childhood, which in turn are known predictors of cardio-metabolic outcomes in adult life. The potential public health and clinical implications of our findings are worth noting. First, identification of developmental BMIz trajectories in the first 2 years may be helpful in identifying high-risk groups. Second, the assessment of prenatal predictors of BMIz trajectories may enhance our understanding of etiologic pathways of cardio-metabolic disease. Lastly, the predictors of early childhood BMIz trajectories, especially modifiable ones (e.g., pre-pregnancy BMI and gestational weight gain), may help in developing effective preventive clinical and public health interventions for cardio-metabolic disease. Future follow-up of our cohort will be important to assess whether the associations we observed persist later in life.

## Electronic supplementary material

Supplementary Information
