# Supplementary material for: Body mass index trajectories in the first two years and subsequent childhood cardio-metabolic outcomes: a prospective multi-ethnic Asian cohort study
Source: Sci Rep. 2017 Aug 21;7:8424. doi: 10.1038/s41598-017-09046-y (PMC5567284; doi:10.1038/s41598-017-09046-y)
Supplement: Supplementary file 1 — Supplementary Information [file 41598_2017_9046_MOESM1_ESM.pdf]

Body mass index trajectories in the first two years and subsequent childhood cardio-metabolic outcomes: a prospective multi-ethnic Asian cohort study

Izzuddin M Aris[1,*], Ling-Wei Chen[2], Mya Thway Tint[3], Wei Wei Pang[3], Shu E Soh[1], Seang-Mei Saw[4], Lynette Pei-Chi Shek[2], Kok-Hian Tan[5], Peter D Gluckman[1, 6], Yap-Seng Chong[1, 3], Fabian Yap[7,8,9], Keith M Godfrey[10], Michael S Kramer[3,11], Yung Seng Lee[1, 2, 12]

Author affiliations
[1] Singapore Institute for Clinical Sciences, Agency for Science, Technology and Research
[2] Department of Paediatrics, Yong Loo Lin School of Medicine, National University of Singapore
[3] Department of Obstetrics and Gynaecology, Yong Loo Lin School of Medicine, National University of Singapore
[4] Saw Swee Hock School of Public Health, National University of Singapore
[5] Department of Obstetrics and Gynaecology, KK Women's and Children's Hospital
[6] Liggins Institute, University of Auckland, Auckland, New Zealand
[7] Department of Paediatrics, KK Women's and Children's Hospital
[8] Duke-NUS Medical School, Singapore
[9] Lee Kong Chian School of Medicine, Nanyang Technological University, Singapore.
[10] MRC Lifecourse Epidemiology Unit and NIHR Southampton Biomedical Research Centre, University of Southampton and University Hospital Southampton NHS Foundation Trust
[11] Departments of Pediatrics and of Epidemiology, Biostatistics and Occupational Health, Faculty of Medicine, McGill University
[12] Khoo Teck Puat-National University Children's Medical Institute, National University Health System, Singapore

Address reprints and correspondence to:
Izzuddin M ARIS. Singapore Institute for Clinical Sciences, Agency for Science, Technology and Research, Singapore. Brenner Centre for Molecular Medicine, 30 Medical Drive Singapore 117609. Tel: +65-6601 5817; Email: Izzuddin_Aris@sics.a-star.edu.sg

Supplemental Figure 1: GUSTO recruitment flowchart

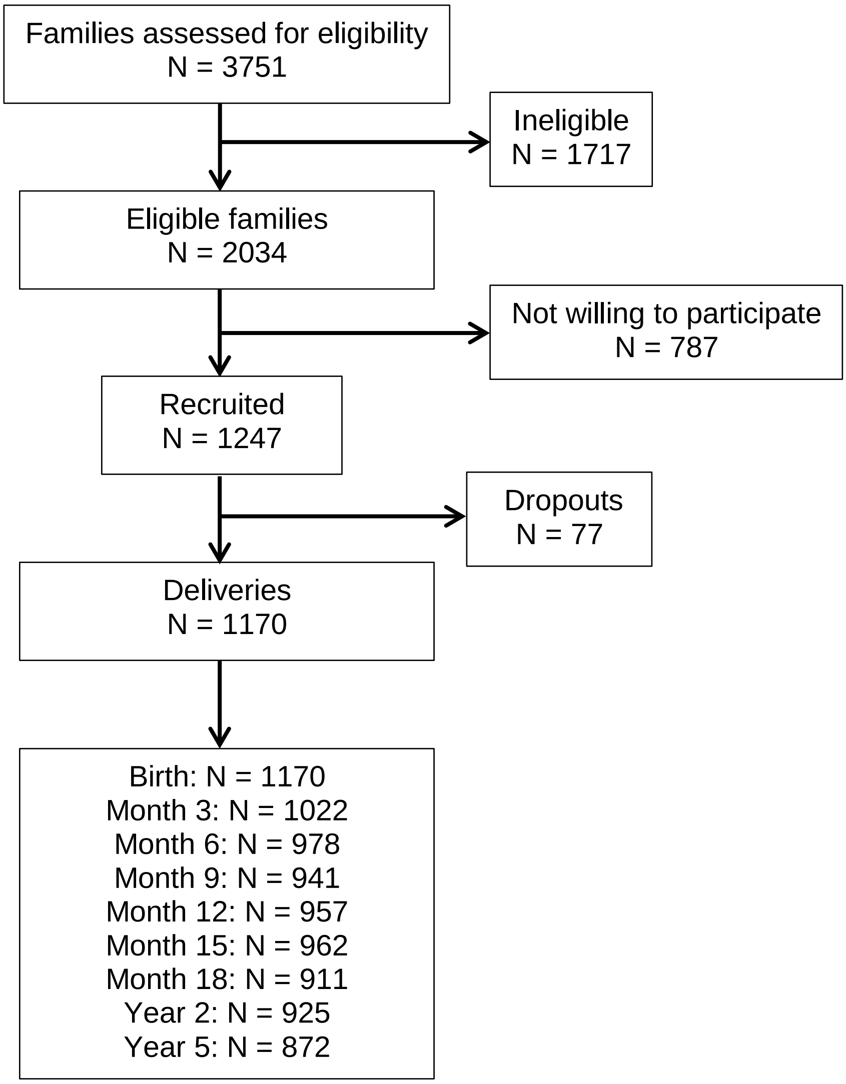

Supplemental Figure 2: BMIz trajectories of children with no missing BMIz data in the first 2 years (n=536)

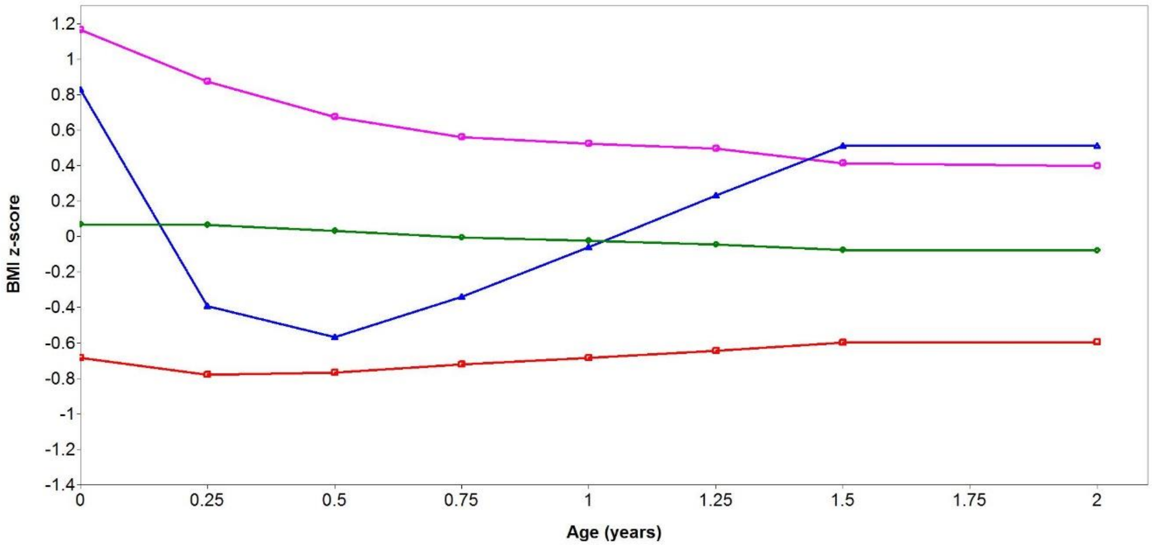

<u>Legend</u>

Stable low BMIz (n=76)
Normal trajectory (n=379)
Stable high BMIz (n=58)
Rapid BMIz gain after 3 months (n=23)

Supplemental Figure 3: Individual trajectory patterns of children belonging to the rapid BMIz gain after 3 months trajectory

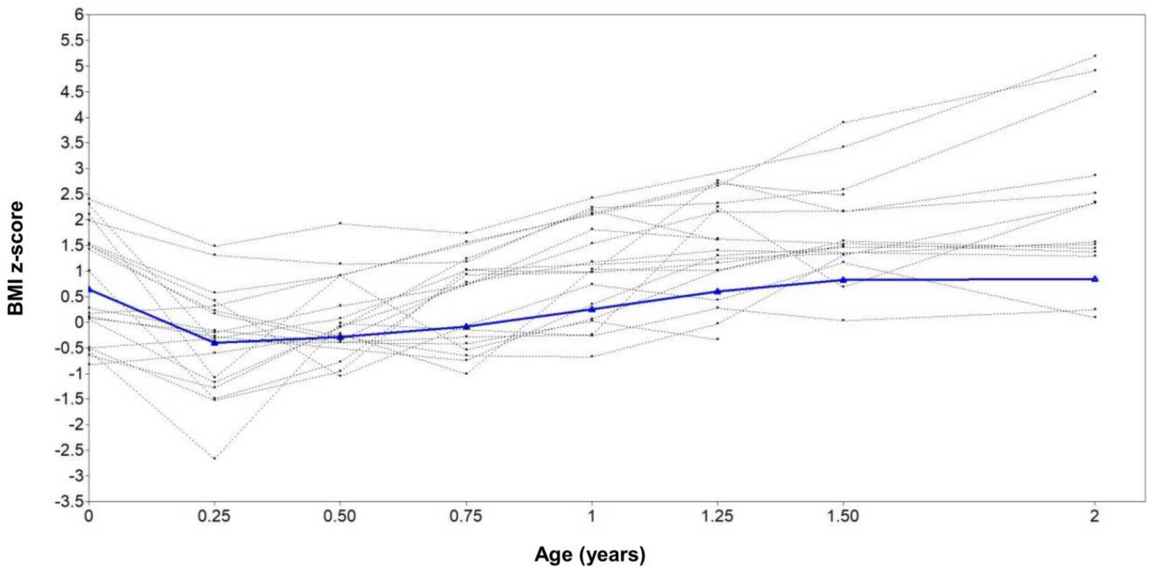

Supplemental Table 1: Frequency and proportion of missing BMIz data at each time point, and across all time points in the first 2 years

**Missing BMIz data at each timepoint**	**n**	**%**
Birth	0	0.0
Month 3	148	12.6
Month 6	192	16.4
Month 9	229	19.6
Month 12	213	18.2
Month 15	208	17.8
Month 18	259	22.1
Month 24	245	20.9
**Missing BMIz data across all timepoints**		
0	536	45.8
1	282	24.1
2	119	10.2
3	62	5.3
4	25	2.1
5	30	2.6
6	30	2.6
7	86	7.3

Supplemental Table 2: Model fit indices for latent-class growth mixture modelling of BMI z-scores in the first 2 years of life

Number of subgroups	Bayesian Information Criterion	Bootstrapped likelihood-ratio test p-value	Average posterior probability (min-max)	Number of subjects per subgroup
2	17892.483	< 0.001	0.819 - 0.847	989/181
3	17813.559	< 0.001	0.805 - 0.843	912/222/36
4	17612.72	0.012	0.748 - 0.790	857/155/100/58
5	17632.501	0.333	0.748 - 0.804	868/152/98/18/34

Supplemental Table 3: Cross-tabulation of BMIz trajectory group assignments in the entire study sample and in those with no missing BMIz data

Entire sample[a] / Completed cases	Stable low BMIz[a]	Normal BMIz[a]	Stable high BMIz[a]	Rapid BMIz gain after 3 months[a]	Row Total
**Stable low BMIz[b]**	76	0	0	0	76
**Normal BMIz[b]**	0	379	0	0	379
**Stable high BMIz[b]**	0	0	58	0	58
**Rapid BMIz gain after 3 months[b]**	0	0	0	23	23
**Column Total**	76	379	58	23	536

[a] Top most header indicates the trajectory patterns derived in the entire sample.
[b] Left-most grid indicates the trajectory patterns derived using the “completed cases” (i.e., children with no missing BMIz data in the first 2 years). The number of subjects for each trajectory pattern is indicated as row total.

Supplemental Table 4: Corresponding BMI percentile at age 2 years for each BMIz trajectory group

BMIz trajectories	Corresponding BMI percentile at age 2 years
**Stable low BMIz (n=155)**	10
**Normal BMIz trajectory (n=857)**	45
**Stable high BMIz (n=100)**	80
**Rapid BMIz gain after 3 months (n=58)**	94

Supplemental Table 5: Booking BMI as a predictor BMI z-score trajectory subgroups in the first 2 years of life

Booking BMI (per unit SD increase)	Odds ratio	95% CI	
		Low	High
Stable low-weight	0.70	0.57	0.88
Normal trajectory	1.00	-	-
Stable high-weight	1.51	1.20	1.92
Rapid weight gain	1.57	1.17	2.12

Other co-variates adjusted in the model include maternal income level, parity, GA at delivery, ethnicity, gestational weight gain and height

Supplemental Table 6: Estimated mean and standard deviations of cardio-metabolic measures in children of the stable high BMIz and rapid BMIz gain trajectories

	Stable high BMIz	Rapid BMIz gain	p value
**Waist-to-Height Ratio (n=864)[a]**	0.50 ± 0.01	0.52 ± 0.01	0.01
**Sum of skinfolds (n=820)[a]**	0.36 ± 0.21	0.70 ± 0.33	<0.01
**Fat-mass index (n=247)[a]**	0.18 ± 0.43	0.90 ± 0.37	<0.01
**Lean-mass index (n=247)[a]**	0.53 ± 0.24	0.44 ± 0.36	0.39
**Systolic blood pressure[b] (n=757)[a,b]**	100.1 ± 1.5	102.7 ± 2.4	0.16
**Diastolic blood pressure[b] (n=757)[a,b]**	59.4 ± 0.9	59.7 ± 0.9	0.97

[a] Estimates represent means and standard deviations adjusted for maternal income level, ppBMI, height, GWG, parity, GA at delivery, breastfeeding, child ethnicity, sex and exact age at measurement

[b] Additionally adjusted for maternal blood pressure at 26-28 weeks of gestation

Supplemental Table 7: Explained variance of BMIz trajectories in the first 2 years of life and static BMIz at age 2 years predicting cardio-metabolic outcomes at age 5-years

**Outcome at 5-years**	**Explained variance**		
	**Covariates only**[a]	**w/ BMIz trajectories**	**w/ static BMIz at 2-years**
**Waist-to-Height Ratio (n=864)**	6.5%	15.3%	15.8%
**Sum of skinfolds (n=820)**	8.2%	14.0%	16.3%
**Fat-mass index (n=247)**	8.8%	26.5%	16.9%
**Lean-mass index (n=247)**	9.4%	17.6%	14.6%
**Systolic blood pressure**[b] **(n=757)**	2.6%	3.4%	3.2%
**Diastolic blood pressure**[b] **(n=757)**	2.7%	3.5%	2.9%
	**Area under ROC curve**		
**Obesity (n=65/872)**	0.71	0.80	0.77
**Prehypertension (n=92/757)**	0.59	0.61	0.61

[a] Covariates only model includes: maternal income level, ppBMI, height, GWG, parity, GA at delivery, breastfeeding, child ethnicity, sex and exact age at measurement
[b] Additionally adjusted for maternal blood pressure at 26-28 weeks of gestation
